# Age-dependent effect of APOE and polygenic component on Alzheimer's disease

**DOI:** 10.1016/j.neurobiolaging.2020.04.024

**Published:** 2020-09

**Authors:** Eftychia Bellou, Emily Baker, Ganna Leonenko, Matthew Bracher-Smith, Paula Daunt, Georgina Menzies, Julie Williams, Valentina Escott-Price, the Alzheimer's Disease Neuroimaging Initiative

**Affiliations:** a UK Dementia Research Institute at Cardiff University, Cardiff, United Kingdom; b UK Medical Research Council Centre for Neuropsychiatric Genetics and Genomics, Division of Psychological Medicine and Clinical Neurosciences, Cardiff University, Cardiff, United Kingdom; c Cytox Ltd, Work.life, Core, Manchester, United Kingdom

**Keywords:** Alzheimer's disease, Polygenic risk scores, Age-depending effects, ADNI, UK Biobank

## Abstract

Alzheimer's disease (AD) is a devastating neurodegenerative condition with significant genetic heritability. Several genes have been implicated in the onset of AD with the apolipoprotein E (*APOE*) gene being the strongest single genetic risk loci. Evidence suggests that the effect of *APOE* alters with age during disease progression. Here, we aim to investigate the impact of *APOE* and other variants outside the *APOE* region on AD risk in younger and older participants. Using data from both the Alzheimer's Disease Neuroimaging Initiative and the UK Biobank, we computed the polygenic risk score of each individual informed by the latest genetic study from the International Genomics of Alzheimer's Project. Our analysis showed that the effect of *APOE* on the disease risk is greater in younger participants and reduces as participants' age increases. Our findings indicate the increased impact of polygenic risk score as participants' age increases. Therefore, AD in older individuals can potentially be triggered by the cumulative effect of genes which are outside the *APOE* region.

## Highlights

• Polygenic risk score analysis was used in ADNI and the UK Biobank data sets.
• *APOE*'s effect on Alzheimer's disease risk was greater in the younger group (age<80 years).
• Genes outside *APOE* region could trigger Alzheimer's disease in older ages (age≥80 years).
• No considerable reduction of *APOE* ε4 alleles in ages less than and more than 80 years old.
• Age-specific genetic scores could aid in clinical trials and personalized medicine.

## 1 Introduction

Late-onset Alzheimer's disease (LOAD) is a devastating neurodegenerative condition with evidence suggesting that the pathobiological process underlying Alzheimer's disease (AD) may begin decades before clinical evidence of dementia (Ritchie et al., 2015). The genetic heritability of LOAD is high (79%); however, the etiology is driven by a combination of genetic and environmental factors (Gatz et al., 2006).

A large number of genes have been implicated in the risk of LOAD (Harold et al., 2009, Jansen et al., 2019, Kunkle et al., 2019, Lambert et al., 2013, Marioni et al., 2018, Sims et al., 2017) with the apolipoprotein E (*APOE*) gene being the strongest genetic risk factor (Strittmatter et al., 1993). *APOE* is a cholesterol carrier involved in lipid transport (Hauser et al., 2011) and brain injury (Houlden and Greenwood, 2006); it exists in 3 common alleles—ε2, ε3, and ε4—with a worldwide prevalence of 8.4%, 77.9%, and 13.7%, respectively (Farrer et al., 1997). Numerous studies have demonstrated that *APOE* ε4 is associated with an earlier age of disease onset (Blacker et al., 1997, Bonham et al., 2016, Frisoni et al., 1998, Sando et al., 2008), longevity (Deelen et al., 2019), and the existence of a gene-dosage effect between increasing copies of ε4 and lower age of AD onset (Corder et al., 1993). This age-dependent genetic heterogeneity was also investigated in the Alzheimer's Disease Genetics Consortium (ADGC) data (Lo et al., 2019); the authors found moderate genetic correlation (r_g_ = 0.64) between the 2 age groups (60–79 years vs. 80+ years), supporting the presence of genetic heterogeneity. Moreover, in their study, the heritability explained by single-nucleotide polymorphisms (SNPs) on chromosome 19, which harbors *APOE*, was substantially larger at younger relative to older age.

A number of retrospective studies have shown that the effect of *APOE* does not exert its influence with the same magnitude during the entire period of AD risk, but mainly in the 60–79 years range (Bickeböller et al., 1997, Davidson et al., 2007). Another study tried to longitudinally examine the effect of age and *APOE* ε4 during progression from normal cognition to AD (Bonham et al., 2016). They reported that *APOE* ε4 influences the progression from mild cognitive impairment to AD in all age groups and the risk varies in age, reaching its peak between ages 70 and 75 years and decreasing after the age of 75 years. In addition, in a meta-analysis of *APOE* data, authors examined the association between *APOE* and AD stratified by age and sex in populations with varying ethnicity and they reported the diminishing effect of *APOE* ε4 after the age of 70 years (Farrer et al., 1997). Finally, a different study tested and confirmed the nonproportional hazard of *APOE* on AD with age. The hazard declined at age 80 years for men and 75 years for women, indicating that different age compositions of study cohorts can result in biases on the estimated effect of *APOE* (Liu and Caselli, 2018).

As prediction algorithms are developed, it will be increasingly important to identify clinically healthy individuals who are at risk of AD and develop therapeutic strategies for intervention. Polygenic risk score (PRS) (Purcell et al., 2009) is a widely used approach which combines the effect of a large number of variants, which may not reach genome-wide significance individually. AD PRS has been shown to be strongly associated with disease (Escott-Price et al., 2015) and explains additional AD risk to that of *APOE* alone (Stocker et al., 2018). The analysis of GERAD data by Escott-Price et al. showed that the effect of PRS is on average slightly lower in the younger group than in the older group (Escott-Price et al., 2015 Supplementary Table 5). This evidence suggests that *APOE* may strongly influence AD risk at younger ages, but as the individuals' age increases, the effect of *APOE* is reduced and therefore, other risk variants start to play a more fundamental role in AD risk. These variants reside in known AD biological pathways with enrichment in immune response, lipid processing, cholesterol metabolism, and endosomal vesicle recycling (Jones et al., 2010, Kunkle et al., 2019).

In this study, we investigated the effect of *APOE* and other variants (combined in a genetic score) on the risk of AD and whether this risk differs with age. We used PRS analysis and we assessed the relationship between AD and PRS using data from 2 prospective studies; the Alzheimer's Disease Neuroimaging Initiative (ADNI) (Petersen et al., 2010, Weiner et al., 2010) and the UK Biobank (UKBB) (Sudlow et al., 2015). It should be noted that the ADNI cohort is older than the UKBB; however, the latter contains parental AD phenotypes and parental age that were used for the analysis. Furthermore, the pathway-specific PRSs, for the 9 pathways found to be significantly associated with AD (Kunkle et al., 2019), were also investigated to determine whether AD risk at older ages is altered by a specific biological pathway. Finally, in the UKBB, we examined whether the ε4's effect in older patients could be attenuated by differential survival because the ε4 allele carriers may have shorter life expectancy (Belloy et al., 2019, Deelen et al., 2019), by exploring the proportion of parents with ages more than and less than 80 years stratified by the *APOE* genotype of the participant. The UKBB participants who are homozygous for these alleles were chosen, thus making sure that each of their parents have at least one of the alleles.

## 2 Materials and methods

### 2.1 Samples description

ADNI is a longitudinal study that was developed for the early detection of AD with the use of clinical, genetic, and imaging data (Petersen et al., 2010). The data were collected from 900 participants between ages 55 and 90 years. Initially, participants were followed for 2 to 3 years with repeated imaging scans and psychometric measurements (ADNI1). The study was extended with the addition of new participants (ADNI-GO and ADNI2). Longitudinal data contained information of clinical assessments from the first, baseline visit to the latest available visit with mean follow-up time approximately 5 years. All participants provided written consent. More information can be found at http://adni.loni.usc.edu/.

Data were available for 770 individuals from ADNI1, ADNI2, and ADNI-GO including genetic information and clinical diagnosis. At the first assessment, 47 individuals were diagnosed with AD, 459 were diagnosed with mild cognitive impairment and 262 were nondemented/controls. At the latest assessment, 174 individuals remained stable to AD and 224 remained nondemented/controls. For this study, we used the latest diagnosis to perform PRS analysis on AD diagnosed individuals versus controls, excluding participants who remained stable or progressed to mild cognitive impairment.

The UKBB is a large prospective cohort of approximately 500,000 individuals from the UK containing extensive phenotypic and genotypic data which are still being collected (Sudlow et al., 2015). Participants were recruited between 2006 and 2010 and were aged 39–72 years. A wealth of lifestyle, sociodemographic, medical, and family history information were collected through a computer-based, self-completed questionnaire during the first assessment visit, along with physical measurements, biological samples, and cognitive testing. Participants were also interviewed by a research nurse to determine any diagnosed medical conditions and their medication use. All participants provided signed consent and are able to withdraw this consent at any stage. Additional information can be found at https://www.ukbiobank.ac.uk/.

This study used the UKBB data under UKBB approval for application 15175 “Further defining the genetic architecture of Alzheimer's disease”. 364,236 white British and Irish individuals remained after removing related individuals and those who have since chosen to withdraw from the study. For the present study, participants were identified as being diagnosed with AD based on diagnosis codes across all hospital inpatient records. Diagnoses are coded according to the International Classification of Diseases version-10 (ICD-10). The UKBB is a young cohort and none of the 204 AD cases were older than 80 years; thus, family history was used as a proxy to AD. Self-report of parental history of AD has been proved to be a valid proxy for an AD genetic study (Marioni et al., 2018). There were 1554 and 38,417 UKBB participants with family history of AD for both parents and one parent, respectively.

### 2.2 Genotyping procedures and quality control (QC)

The ADNI samples were genotyped using non-CLIA whole-genome sequencing and Illumina Omni 2.5 M BeadChip array and basic QC was performed. Additional QC checks were performed using PLINK (https://www.cog-genomics.org/plink2/) (Marees et al., 2018). These included exclusion of SNPs with minor allele frequency less than 0.01 and genotype missingness greater than 0.02 and SNPs with Hardy-Weinberg equilibrium *p*-value ≤ 10^−6^. After QC, 7,808,548 SNPs were included in the analyses.

The UKBB contains 35,884,914 imputed SNPs. These imputed data were QCed by removing rare SNPs with minor allele frequency <0.01, SNPs imputed with poor accuracy (INFO ≤0.4), SNPs with posterior probability ≤0.4, SNPs with missing data proportion >0.05, and SNPs which violate Hardy-Weinberg equilibrium with *p* < 10^−6^. After these QC steps, 7,654,308 SNPs remained for the analysis.

### 2.3 Calculation of PRSs

#### 2.3.1 Discovery cohort

The PRS (Purcell et al., 2009) combines the effect of a large number of genetic variants which individually may not reach genome-wide significance. The summary statistics from the largest available genome-wide association study (GWAS) on AD (Kunkle et al., 2019), which is independent from the UKBB sample, were used to generate genetic scores for both ADNI and UKBB participants as the weighted sum of the risk alleles. This GWAS is an extension to the International Genomics of Alzheimer's Project (IGAP) data (Lambert et al., 2013); the results were meta-analyzed to an additional replication set, thus increasing sample size to N = 63,926 (stage 1). These summary statistic data were chosen as they are independent from the UKBB sample, and although the ADNI data are part of the IGAP study, the overlap does not significantly affect the PRS prediction (Leonenko et al., 2019). More specifically, a total of 441 ADNI participants, accounting for 0.7% of the Kunkle et al., 2019 study, were part of the Alzheimer's Disease Genetics Consortium (Naj et al., 2011) which was used by the IGAP study. Leonenko et al performed PRS analysis, adjusting for this overlap by using 1000 simulations. They modeled the variation attributed to the exclusion of 441 samples from the Lambert et al., 2013 summary statistics (0.8%) to account for potential biases and showed that prediction accuracy was not affected (Leonenko et al., 2019).

#### 2.3.2 Controlling for linkage disequilibrium

The standard PRS approach assumes independence between SNPs; therefore, data were pruned for linkage disequilibrium (LD). ADNI data were LD-pruned with PLINK (http://zzz.bwh.harvard.edu/plink/) (Purcell et al., 2007) by retaining the variant with the smallest *p*-value from each LD block and excluding variants with r^2^ > 0.1 in 1000-kb window. The imputed UKBB data were LD-pruned in the same way, using a random sample of 1000 individuals with “best guess genotypes” computed from allele “dosages”.

#### 2.3.3 Generation of genetic scores

PLINK was used to construct the PRSs for each participant using as significance threshold the *p*-value p_T_ ≤ 0.5 because of its prediction accuracy (Escott-Price et al., 2015). 255,026 and 240,984 SNPs were used to derive the PRS excluding *APOE*, in ADNI and UKBB, respectively.

#### 2.3.4 Identification of predictors and confounders

In the current analyses, *APOE* ε2 and ε4 and PRSs without the *APOE* region were the main predictors. In both ADNI and the UKBB, the effects of *APOE* ε2 and ε4 were estimated in the data using univariate logistic regression on disease status and were directly included to the PRS, while excluding the *APOE* region (chromosome 19:44.4Mb 19:46.5Mb).

The genetic scores were adjusted for age, sex, and principal components (PCs) and then standardized. In ADNI, the age of the participants at the last assessment visit was used along with eight PCs. In the UKBB, the age at the parents' death or the last recorded age if they were alive and 15 PCs were used. In both data sets, the sex of the participants in the study was used. Finally, an additional analysis was performed using PRSs adjusted for PCs only and including age and sex as predictors in the regression models as both variables have predictive value for AD (Escott-Price et al., 2015). To investigate the contribution of *APOE* and PRS at the specific age ranges, individuals were grouped in those of less than 80 years and those of more than 80 years of age.

#### 2.3.5 Generation of pathway-specific genetic scores

We produced a pathway-specific PRS in both data sets for each of the 9 pathways found to be associated with AD (Kunkle et al., 2019). Genes from the 9 Kunkle pathways were mapped to GENCODE (v29) (Harrow et al., 2012) data, where only known, protein coding genes were retained. The pathways were defined by all SNPs which reside between the start and end base position of genes in the pathway (see Supplementary Table 1). The 9 significantly associated pathways are protein-lipid complex assembly, regulation of beta-amyloid formation, protein-lipid complex, regulation of amyloid precursor protein catabolic process, reverse cholesterol transport, protein-lipid complex subunit organization, plasma lipoprotein particle assembly, tau protein binding, and activation of immune response. It should be noted that in contrast to the whole-genome PRS, pathway-specific analysis models the *APOE* region rather than the genotype of the 2 *APOE* SNPs (rs7412 and rs429358). The reason for this is to capture additional genetic variation by the pathways and thus, increase the power. For example, the “activation of immune response” pathway contains the *RELB* gene which is in the *APOE* region but does not contain the *APOE* gene.

### 2.4 Statistical analysis

#### 2.4.1 Descriptive analysis

Normally distributed variables were presented using means with standard deviations.

#### 2.4.2 Association of PRSs with disease status

The association of PRSs with AD status in ADNI was examined using logistic regression including the following predictors: a) the direct count of *APOE* ε4 and ε2 alleles and b) PRS without the *APOE* region. A Poisson regression model was used in the UKBB data to assess the relationship between PRS and parental AD, as the AD was coded as 0, 1, 2 indicating the number of parents with the disease, including the same predictors. A likelihood ratio test was used to determine whether a) *APOE* and the PRS excluding *APOE* both show an association with AD and b) the PRS excluding *APOE* explains any additional variation. Finally, in the UKBB, the same analysis was also performed for maternal and paternal AD separately using logistic regression analysis.

## 3 Results

### 3.1 Association of AD PRS with disease risk stratified by age

Table 1 depicts the association results between AD status and PRSs in ADNI after adjusting for PCs, participants' sex and age at latest assessment visit. A likelihood ratio test indicated that PRS significantly improves the prediction accuracy of the model in all 3 groups (all, participants younger than 80 years, and participants older than 80 years) over and above *APOE* (*p* = 6.08 × 10^−14^, *p* = 6.62 × 10^−5^, and *p* = 1.95 × 10^−11^, respectively). Examining the effect sizes of *APOE* only and PRS excluding the *APOE* region, the β_APOE_ is smaller in the older group (β = 0.78, SE = 0.174, *p* = 7.08 × 10^−6^) as compared with the younger group (β = 1.151, SE = 0.194, *p* = 3.01 × 10^−9^) in which β_PRS_ is larger (β = 1.456, SE = 0.229, *p* = 1.92 × 10^−10^ in ages ≥80 years vs. β = 0.677, SE = 0.169, *p* = 1.99 × 10^−5^ in ages <80 years). This suggests that *APOE* is more important for AD development in individuals younger than 80 years and above that age, a polygenic component has a significantly higher contribution over and above *APOE* alone. As age and sex have predictive value for AD (Escott-Price et al., 2015), analysis was performed including these 2 variables in each age strata in addition to *APOE* and PRS, with results remaining the same (results not shown).

**Table 1:** Association results between disease status and polygenic risk scores, in ADNI data set

Phenotype	Age	N	*APOE* (ε4 + ε2)				PRS (excl. *APOE* region) (*p*_T_ ≤ 0.5)				*APOE* + PRS (*p*_T_ ≤ 0.5)	
			β	SE	*p*	AUC	β	SE	*p*	AUC	*p*	AUC
AD (latest assessment)	All	AD = 174 NL = 224	0.951	0.126	4.10 × 10^−14^	0.723	1.066	0.138	1.34 × 10^−14^	0.747	<1 × 10^−350^	0.807
	<80	AD = 87 NL = 125	1.151	0.194	3.01 × 10^−9^	0.744	0.677	0.169	1.99 × 10^−5^	0.677	1.75 × 10^−10^	0.798
	≥80	AD = 87 Nl = 99	0.780	0.174	7.08 × 10^−6^	0.667	1.456	0.229	1.92 × 10^−10^	0.811	7.04 × 10^−11^	0.825

Logistic regression was performed. The models were adjusted for PCs, participants' age at last assessment and sex.

Genetic variants with *p*-value *p*_T_ ≤ 0.5 from Kunkle et al. (2019) summary statistics were used to calculate the genetic score.

Key: AD, β, beta coefficient (effect size); Alzheimer's disease; *APOE*, apolipoprotein E; AUC, area under the curve; excl., excluding; N, sample size; NL, nondemented/controls; *p*, *p*-value; *p*_T,_*p*-value with threshold T ≤ 0.5; PRS, polygenic risk score; SE, standard error.

The association between AD PRS and parental AD using the UKBB data is shown in Table 2. Parental AD shows that PRS excluding *APOE* is associated with AD over the effect of *APOE*. When examining the effect sizes for *APOE*, it is seen that the effect size is smaller in the older group (β = 0.208, SE = 0.005, *p* < 1 × 10^−350^) compared with the younger group (β = 0.348, SE = 0.009, *p* < 1 × 10^−350^). The *p*-values differ between the parental AD age groups; this is due to the differences in sample size. Therefore, it is appropriate to interpret the β values here.

**Table 2:** Association results between parental AD and polygenic risk scores in the UK Biobank data

Phenotype	Age	N	*APOE* (ε2 + ε4)				PRS (excl. *APOE* region) (p_T_ ≤ 0.5)				*APOE* + PRS (*p*_T_ ≤ 0.5)	
			β	SE	*p*	AUC	β	SE	*p*	AUC	*p*	AUC
Parental AD	All	BP = 1554 OP = 38,417 NP = 284,110	0.242	0.004	<1 × 10^−350^	0.576	0.031	0.005	2.4 × 10^−10^	0.501	<1 × 10^−350^	0.573
	<80	BP = 326 OP = 10,755 NP = 213,877	0.348	0.009	<1 × 10^−350^	0.591	0.037	0.009	0.00008	0.511	<1 × 10^−350^	0.598
	≥80	BP = 1228 OP = 27,662 NP = 70,233	0.208	0.005	<1 × 10^−350^	0.569	0.030	0.006	1.7 × 10^−7^	0.510	6.0 × 10^−314^	0.576

Poisson regression was performed. The models were adjusted for PCs, parental age and participants' sex. Genetic variants with *p*-value *p*_T_ ≤ 0.5 from Kunkle et al. (2019) summary statistics were used to calculate the genetic score. The parental age was either the age at death or the last recorded age if alive.

Key: N, sample size; β, beta coefficient (effect size); AD, Alzheimer's disease; *APOE*, apolipoprotein E; AUC, area under the curve; BP, both parents have AD; excl., excluding; NP, neither parent has AD; OP, one parent has AD; *p*, *p*-value; p_T,_*p*-value with threshold T ≤ 0.5; PRS, polygenic risk score; SE, standard error.

The β values between ADNI and UKBB are comparable, with the effect of *APOE* showing consistent results between age groups across both cohorts. The effect sizes for parental AD in UKBB are much smaller compared with ADNI because the association between PRS and a proxy to AD is being considered. In the UKBB, the effect sizes between the different age groups for PRS excluding *APOE* are very similar (all: β = 0.031, <80: β = 0.037, ≥80: β = 0.030).

The parental AD association was surprisingly high when considering that the individual was used to predict parental outcome, where only half of the genome is shared among them. The corresponding results for the models which included age and sex as predictors were similar (results not shown).

Table 3 displays the effect of *APOE* and PRS (excluding *APOE*) on maternal and paternal AD separately. It can be observed that the effect sizes in mothers is slightly higher than in fathers and this effect was consistent for both *APOE* and PRS excluding *APOE.* Note that these effect sizes are from a logistic regression model and are therefore not directly comparable with the results in Table 2.

**Table 3:** Association results between parental AD (split into maternal and paternal AD) and PRSs in the UK Biobank

Phenotype	Age	*APOE* (ε2 + ε4)	PRS (excl. *APOE* region) (*p*_T_ ≤ 0.5)
		β	β
Maternal AD	All	0.275	0.035
Paternal AD		0.240	0.031
Maternal AD	<80	0.404	0.044
Paternal AD		0.297	0.030
Maternal AD	≥80	0.273	0.038
Paternal AD		0.225	0.034

Logistic regression was performed. The models were adjusted for PCs, parental age, and participants' sex. Genetic variants with *p*-value p_T_ ≤ 0.5 from Kunkle et al. (2019) summary statistics were used to calculate the genetic score. The parental age was either the age at death or the last recorded age if alive.

Key: β, beta coefficient (effect size); AD, Alzheimer's disease; *APOE*, apolipoprotein E; excl., excluding; *p*_T,_*p*-value with threshold T ≤ 0.5; PRS, polygenic risk score.

### 3.2 Age of parents with the *APOE* alleles

The parental age of the UKBB participants ranged between 60 and 107 years. Selecting UKBB participants who are homozygous for *APOE* (ε4ε4, ε3ε3, ε2ε2) alleles, we ensured that each parent had at least one *APOE* allele (ε4, ε3, and ε2, respectively). The mothers were 5–6 months older than the fathers within each *APOE* stratum when their age was last recorded. The last recorded age was similar between the mothers and the fathers within the 3 *APOE* groups. The age at death was about 4 years earlier for the fathers than for the mothers (see Table 4). Furthermore, we observed a 12-month earlier age at death for ε4 carriers as compared with ε2 carriers in either male of female parents, whereas the ε3 carriers were in between.

**Table 4:** Parental last recorded age and age at death for participants homozygous to *APOE* alleles in the UK Biobank

*APOE* genotype of UKBB participants	N	Mothers' last recorded age	Fathers' last recorded age	Mothers' age at death	Fathers' age at death
		Mean (sd)	Mean (sd)	Mean (sd)	Mean (sd)
ε4ε4	10,563	78.2 (7.8)	77.9 (7.1)	74.3 (12.7)	70.4 (12.8)
ε3ε3	258,659	79.1 (8.2)	78.4 (7.5)	74.7 (13.2)	70.8 (13.0)
ε2ε2	2808	78.9 (8.3)	78.3 (7.7)	75.4 (12.7)	71.6 (12.6)

If parent was alive, the last recorded age was used. Otherwise, the age of death of the parent was used.

Key: APOE, apolipoprotein E; N, number of samples; sd, standard deviation; UKBB, UK Biobank.

After splitting the parents into age groups less than and more than 80 years old (as before), we did not observe a substantial difference in proportions of mothers or fathers of the UKBB participants with either of *APOE* alleles (Table 5). A slight decrease in the number of ε4 carriers was observed at the age of 86+ years (see Supplementary Fig. 1).

**Table 5:** Proportion of parents of participants homozygous to *APOE* alleles in the UKBB stratified by age groups

*APOE* genotype in the UKBB participants	Fathers (proportion)		Mothers (proportion)	
	<80	≥80	<80	≥80
ε4ε4	0.040	0.036	0.041	0.036
ε3ε3	0.950	0.953	0.949	0.953
ε3ε3	0.010	0.011	0.010	0.011

If parent was alive, the last recorded age was used. Otherwise, the age of death of the parent was used.

Key: *APOE*, apolipoprotein E; UKBB, UK Biobank.

### 3.3 Pathway-specific PRS analysis

We have also estimated pathway-specific PRSs for the 9 pathways which were associated with AD in Kunkle et al. GWAS (Kunkle et al., 2019) and examined the association with the risk of AD phenotypes. All pathway analyses were performed with and without *APOE*.

**Fig. 1:**
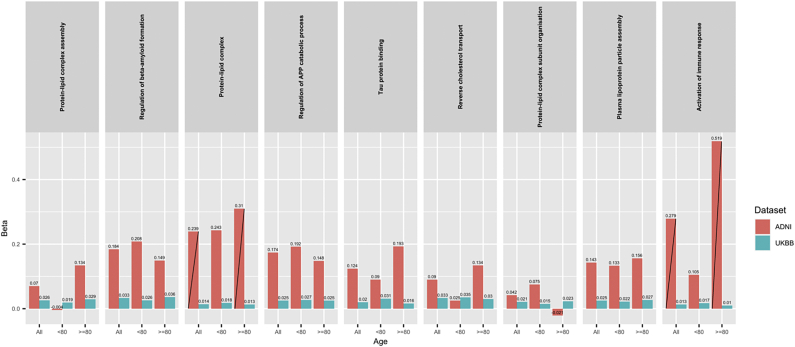
Association of pathway-specific genetic scores (excluding *APOE* region) with the risk of Alzheimer's disease. Depiction of ADNI (red) and UKBB (blue) data for all individuals and stratified by age. Bars crossed with a black line depict statistically significant results. Abbreviations: ADNI, Alzheimer's Disease Neuroimaging Initiative; UKBB, UK Biobank. (For interpretation of the references to color in this figure legend, the reader is referred to the Web version of this article.)

The results can be seen in Fig. 1 and in Supplementary Tables 2 and 4 for ADNI and UKBB data sets, respectively; with SEs and AUC shown in Supplementary Tables 3 and 5, respectively. All effect sizes were reduced in the UKBB as compared with ADNI because of the use of a “proxy” for AD rather than directly assessed AD.

In ADNI data, all pathways in all age groups (all, <80, ≥80), except the activation of immune response, were associated with the risk of AD; however, the association was driven by the *APOE* region (see Supplementary Tables 2 and 3). Only “protein-lipid complex” remained significantly associated with AD after removing the *APOE* region (β = 0.239, *p* = 0.021 for all participants and β = 0.310, *p* = 0.043 for participants older than 80 years), with PRS marginally adding to the genetic risk stratification above and beyond *APOE*; this is consistent with the results presented in a previous study (Leonenko et al., 2019). As before, the effect sizes for *APOE* were larger in the younger group for all 9 pathways compared with the older group, supporting the increased effect of *APOE* in younger ages. Similar results were found when including both age and sex as predictor variables in the regression models (results not shown).

The “activation of immune response” pathway, which does not contain *APOE* gene, showed a clear association in full ADNI sample (β = 0.279, *p* = 0.008) and an even stronger association in the older age group (β = 0.519, *p* = 0.001), whereas in the younger group the association was not significant (*p* = 0.458).

In the UKBB, all pathways showed a significant association with parental AD for both *APOE* region and PRS excluding *APOE* region except for the “activation of immune response” and “protein-lipid complex subunit organization” pathway for which the results did not remain significant after splitting the participants into age groups (Supplementary Tables 4 and 5). The increased number of significant results was due to the much larger sample size in the UKBB compared with ADNI; however, the quantity of significant results is noteworthy when parental AD is used as a proxy and therefore effect sizes are small. Again, the effect sizes in parental AD for *APOE* were larger in the younger group compared with the older group, supporting the increased *APOE* effect in younger individuals. The effect sizes for PRS excluding *APOE* are small, and therefore, it is difficult to compare them across groups; 4 of the 9 pathways have a slightly larger effect size in the older group compared with the younger group. Similar results are shown when using age and sex as predictors in the model.

The “activation of immune response” pathway association in the UKBB did not show a clear pattern as in the ADNI data. Although in the group of all parents, the association of this pathway was statistically significant (β = 0.013, *p* = 0.0068), when split by age, the *p*-values become nonsignificant (*p* = 0.060 and *p* = 0.073, in the younger and older groups, respectively). The effect sizes of the parental AD association were similar in all 3 groups (all, <80, ≥80); however, these are quite reduced when compared with the ADNI results. As pointed out earlier, this is likely to be due to the “association via proxy” analyses and as a consequence, the reduced power of the analyses which in turn potentially explains the absence of clear pattern, leaving the “activation of immune response” pathway association results inconclusive (non-replicated) in this analysis.

Finally, for both ADNI and the UKBB, the β values across both age groups were compared. There was strong evidence for a difference in βs between the younger and older groups in all pathways based on *APOE* across both data sets, but there was no difference observed between the βs for each age group for the PRS association excluding *APOE*.

## 4 Discussion

It is known that both age and PRS (including *APOE*) contribute to liability to AD, but their etiological relationship has not been fully elucidated. We evaluated an additive model whereby risk of AD requires less contribution from common SNPs in younger individuals. Our study benefits from the use of the publicly available and well-studied ADNI data and the confirmation of results in the UKBB data using family history as a proxy of AD.

Our study not only shows that the effect of *APOE* is stronger in the younger group compared with the older group, aged at least 80 years, but it suggests that the effect of the polygenic component at the latter age group is significantly greater than *APOE* alone. This implies that potentially other/additional biological mechanisms of AD development can be identified by the polygenic component over and above *APOE* in the older individuals. Therefore, the age-varying effect of *APOE* and the polygenic component of AD should be taken into consideration in both clinical and scientific settings. The complex interplay between the genetic architecture, sex, and age of patients indicates the incorporation of these risk factors in both treatment planning and enrollment in clinical trials for identifying cognitively normal *APOE* ε4 carriers progressing to AD (Riedel et al., 2016). The greater effect of PRS in older patients may aid in stratifying individuals for precision medicines, and individuals whose AD is predominantly influenced by *APOE* may require different treatments to those effected by the polygenic component of remaining variants.

The work presented in this article supports findings from previous studies. The heterogeneity of the polygenic architecture across age in AD was demonstrated by Lo et al. The authors showed that the heritability explained by chromosome 19 was significantly larger in the younger participants (Lo et al., 2019). Similar results have also been presented in previous studies by Escott-Price (Escott-Price et al., 2015, Escott-Price et al., 2017). Moreover, a number of studies have suggested differences in magnitude of the effect of *APOE* during the entire period of AD risk, with *APOE* ε4 reaching its peak effect between the age of 60–79 years (Bickeböller et al., 1997, Bonham et al., 2016).

Our pathway association results were inconclusive in this analysis. For both data sets, the effect sizes for *APOE* were larger in all 9 pathways in the younger group compared with the older cohort; whereas, the effect sizes of PRS excluding the *APOE* region were larger in 6 of 9 pathways in ADNI and in 4 of 9 pathways in the UKBB. Furthermore, the “activation of immune response” pathway association in the UKBB did not show a clear pattern as in the ADNI data, likely due to the reduced statistical power.

The findings of this study have to be seen in light of some limitations. ADNI is an amnestic clinical population with no reported comorbidities to be adjusted for in the analysis. Moreover, the sample size is relatively low, especially when split into age groups. To address the latter limitation, the UKBB was used for replication purposes; however, the UKBB data are reliant on a proxy for AD (the parental AD). Although the analysis by proxy was been used previously (Marioni et al., 2018), it should be noted that the parental AD has been grouped by the designers of the UKBB with other forms of dementia whose prevalence at different age groups could potentially bias our results. On the same note, the parental AD phenotype used in the present study combines AD/dementia in both parents to reduce multiple testing; therefore, parental sex effects could not be accounted for in our models. We did, however, investigate maternal and paternal AD separately, which showed that effect sizes are consistently higher in mothers compared with fathers. Another potential limitation is the “arbitrary” choice of the age cutoff used for splitting the subjects into groups. Nevertheless, these cutoffs seem to be in line with ages being reported in previous studies when the effect of *APOE* started declining. Furthermore, the decrease in *APOE* frequency in older ages (Frisoni et al., 1998, Murman et al., 1996, Sando et al., 2008) could be due to censoring by the earlier onset of AD in *APOE* ε4 carriers. We tried to address this limitation by splitting the parents into age groups less than and more than 80 years old; however, we did not observe major difference in the proportion of mothers or fathers on the UKBB participants with either of *APOE* alleles. Further investigation is needed to examine the potential existence of survival biases. Another plausible explanation could be that *APOE* ε4 carriers who have not progressed to cognitive impairment during the risk age range of 65–75 years, might possess protective genetic or lifestyle factors that delay that progression.

## 5 Conclusion

In this study, we emphasize that both *APOE* and PRS are predictors of AD risk presenting age-dependent effects on progression to cognitive decline. We show that the effect of *APOE* on AD risk is stronger in individuals with age less than 80 years, whereas PRS contributes more to the risk of AD development in ages more than 80 years. In the older group, the polygenic risk score has shown to be the same or higher compared with the younger group. Age-based risk estimates of the genetic architecture of the disease could aid in clinical trials of disease-modifying treatments and personalized medicine once effective therapies are available.

## Disclosure statement

The authors EBellou, JW, and VEP have collaborated with Cytox Ltd through their Innovate UK grant funding. PD is an employee of Cytox Ltd.

## CRediT authorship contribution statement

**Eftychia Bellou:** Conceptualization, Methodology, Software, Formal analysis, Resources, Writing - original draft, Visualization. **Emily Baker:** Conceptualization, Methodology, Software, Formal analysis, Resources, Writing - original draft, Visualization. **Ganna Leonenko:** Methodology, Software, Validation, Writing - review & editing. **Matthew Bracher-Smith:** Validation, Data curation, Investigation, Writing - review & editing. **Paula Daunt:** Validation, Writing - review & editing, Funding acquisition. **Georgina Menzies:** Validation, Data curation, Writing - review & editing. **Julie Williams:** Conceptualization, Writing - review & editing, Funding acquisition. **Valentina Escott-Price:** Conceptualization, Investigation, Writing - review & editing, Supervision, Project administration, Funding acquisition.

## Appendix A Supplementary data

Supplementary data

Data Profile
