# Supplementary material for: Age-dependent effect of APOE and polygenic component on Alzheimer's disease
Source: Neurobiol Aging. 2020 Sep;93:69–77. doi: 10.1016/j.neurobiolaging.2020.04.024 (PMC7308803; doi:10.1016/j.neurobiolaging.2020.04.024)
Supplement: Supplementary data [file mmc2.docx]

**Appendix A. Supplementary Information**

**Supplementary Table 1 (Kunkle_pathway_Gene_SNPs.csv file)**

The file contains the nine pathways from (Kunkle et al., 2019) found to be associated with Alzheimer’s disease, along with the list of the protein-coding genes of each pathway (as mapped to GENCODE (v29). Pathways were defined by all SNPs which reside between the start and end base position of genes in the pathway. The APOE column indicates whether the SNP is part of the APOE region (listed as 1) or not (listed as 0).

**Supplementary Figure 1**

Frequencies of e4, e3 and e2 alleles vs deciles of parental age of UKBB participants homozygous for *APOE* alleles. The red line represents the participants homozygous to the APOE ε4 allele, the blue line represents the participants homozygous to the *APOE* ε3 allele and finally, the green line represents the participants homozygous to the *APOE* ε2 allele. The last recorded age was used, if parent was alive, the age of death of the parent was used otherwise.


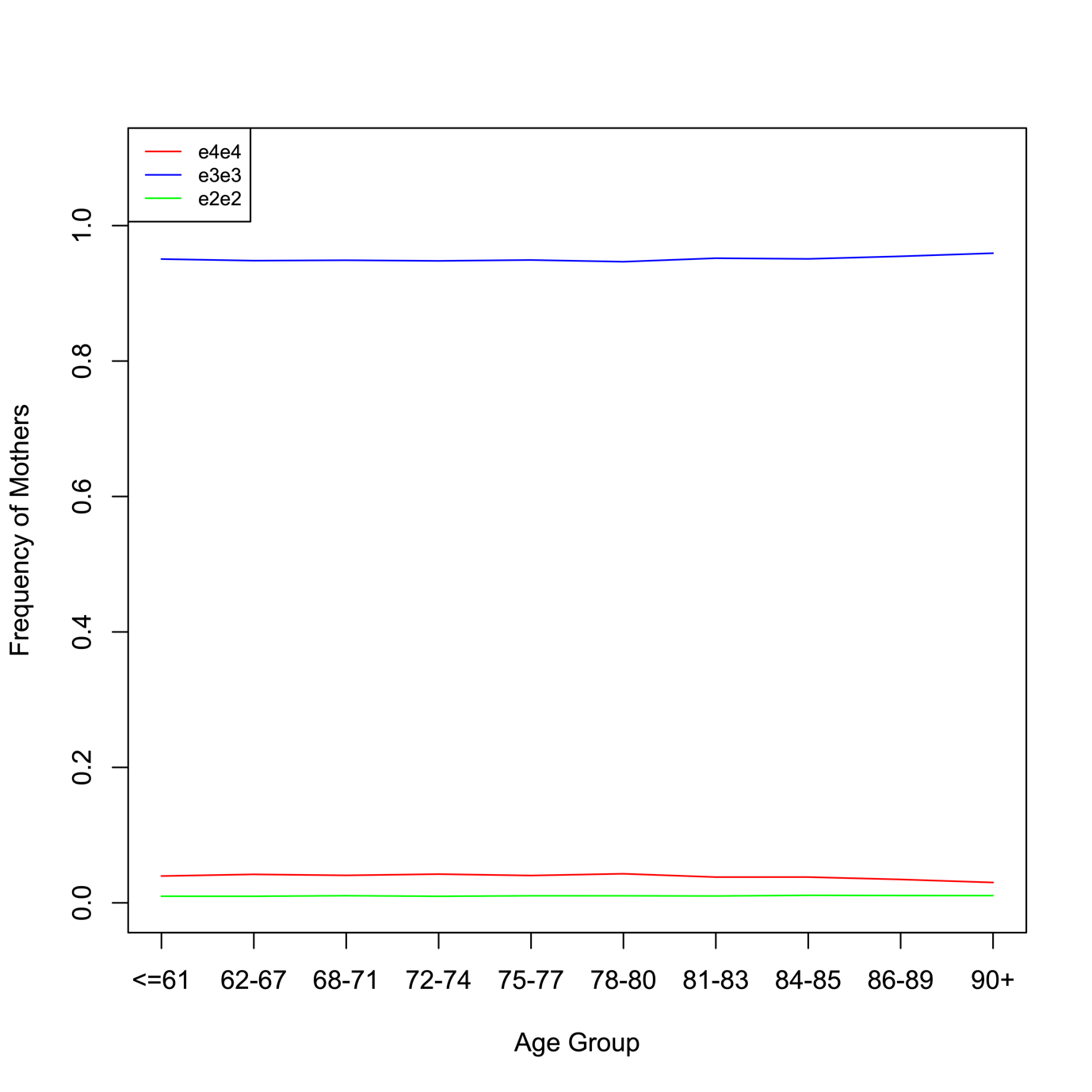


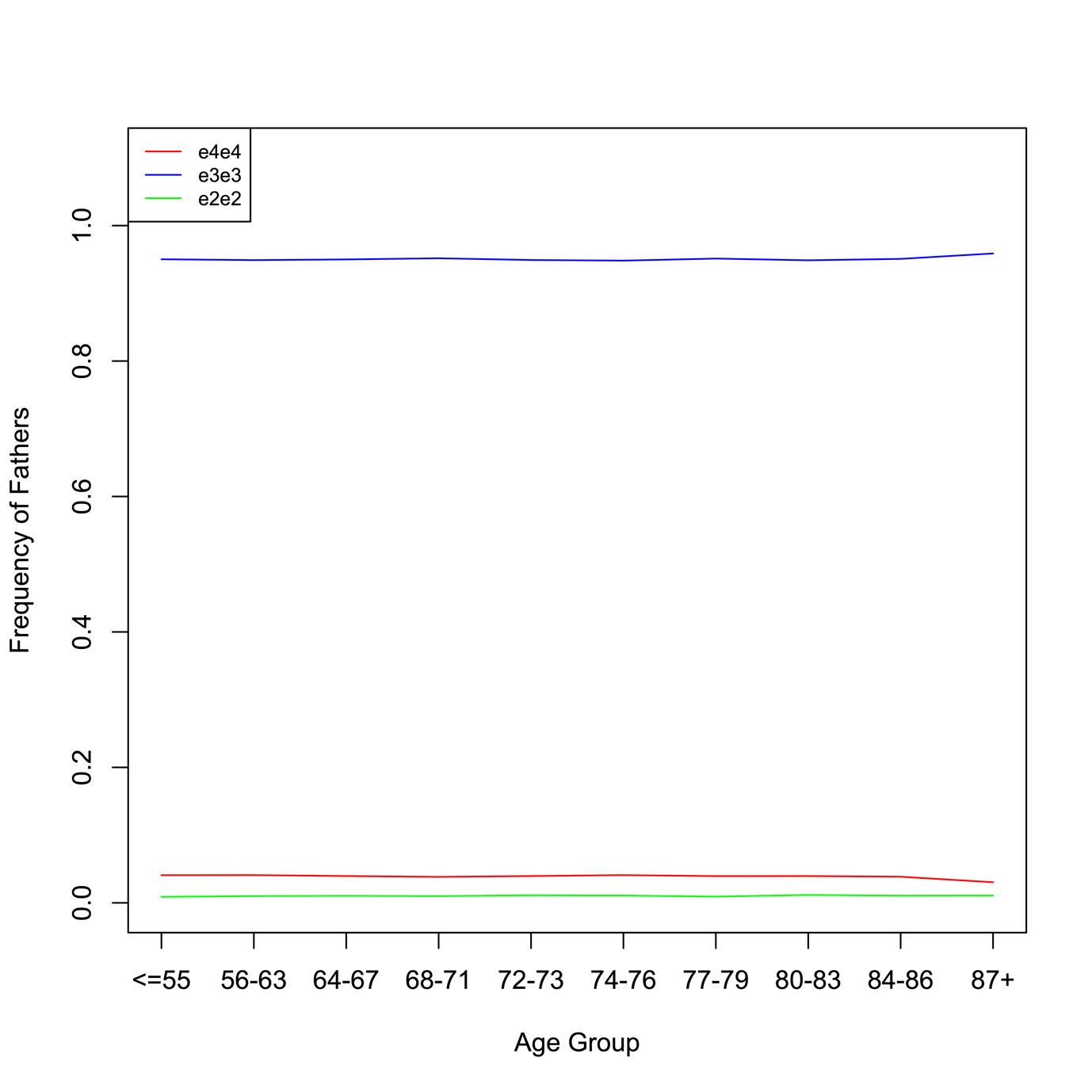


**Supplementary Table 2**

Association results between disease status and Alzheimer’s disease pathway PRS in ADNI data – effect sizes (β) and p-values (P)

| **Phenotype** | **Pathway** | **Age** | ***APOE* region^¥^** | | **PRS (excl. *APOE* region)**  **(p_T_**≤**0.5)** | | ***APOE* + PRS**  **(p_T_**≤**0.5)** | |  |
| --- | --- | --- | --- | --- | --- | --- | --- | --- | --- |
|  |  |  | **β** | **P** | **β** | **P** | | **P** | |
| **AD**  **(latest assessment)** | **Protein-lipid complex assembly** | All | 0.856 | 1.42x10^-11^ | 0.070 | 0.487 | | 7.42x10^-12^ | |
|  |  | < 80 | 1.090 | 2.64x10^-9^ | -0.004 | 0.980 | | 2.37x10^-8^ | |
|  |  | ≥ 80 | 0.598 | 2.47x10^-4^ | 0.134 | 0.367 | | 1.75x10^-4^ | |
|  | **Regulation of beta-amyloid formation** | All | 0.804 | 2.65x10^-11^ | 0.184 | 0.072 | | 3.12x10^-11^ | |
|  |  | < 80 | 1.145 | 6.69x10^-10^ | 0.208 | 0.143 | | 1.61x10^-9^ | |
|  |  | ≥ 80 | 0.417 | 8.15x10^-3^ | 0.149 | 0.317 | | 5.13x10^-3^ | |
|  | **Protein-lipid complex** | All | 0.823 | 8.40x10^-12^ | 0.239 | 0.021 | | 6.06x10^-12^ | |
|  |  | < 80 | 1.096 | 2.34x10^-9^ | 0.243 | 0.089 | | 5.22x10^-9^ | |
|  |  | ≥ 80 | 0.510 | 1.51x10^-3^ | 0.310 | 0.043 | | 3.52x10^-4^ | |
|  | **Regulation of APP catabolic process** | All | 0.804 | 2.65x10^-11^ | 0.174 | 0.089 | | 4.72x10^-11^ | |
|  |  | < 80 | 1.145 | 6.69x10^-10^ | 0.192 | 0.176 | | 2.26x10^-9^ | |
|  |  | ≥ 80 | 0.417 | 8.15x10^-3^ | 0.148 | 0.319 | | 5.48x10^-3^ | |
|  | **Tau protein binding** | All | 0.803 | 3.06x10^-11^ | 0.124 | 0.225 | | 6.41x10^-11^ | |
|  |  | < 80 | 1.155 | 5.1x10^-10^ | 0.090 | 0.542 | | 2.63x10^-9^ | |
|  |  | ≥ 80 | 0.396 | 0.012 | 0.193 | 0.196 | | 5.18x10^-3^ | |
|  | **Reverse cholesterol transport** | All | 0.849 | 1.96x10^-12^ | 0.090 | 0.373 | | 3.27x10^-11^ | |
|  |  | < 80 | 1.091 | 2.24x10^-9^ | 0.025 | 0.861 | | 5.93x10^-8^ | |
|  |  | ≥ 80 | 0.574 | 4.11x10^-4^ | 0.134 | 0.367 | | 3.77x10^-4^ | |
|  | **Protein-lipid complex subunit organization** | All | 0.856 | 1.42x10^-12^ | 0.042 | 0.678 | | 2.68x10^-12^ | |
|  |  | < 80 | 1.089 | 2.64x10^-9^ | 0.075 | 0.591 | | 3.16x10^-9^ | |
|  |  | ≥ 80 | 0.598 | 2.47x10^-4^ | -0.021 | 0.888 | | 4.45x10^-4^ | |
|  | **Plasma lipoprotein particle assembly** | All | 0.804 | 2.65x10^-11^ | 0.143 | 0.160 | | 7.10x10^-11^ | |
|  |  | < 80 | 1.145 | 6.69x10^-10^ | 0.133 | 0.348 | | 2.76x10^-9^ | |
|  |  | ≥ 80 | 0.417 | 8.15x10^-3^ | 0.156 | 0.296 | | 6.04x10^-3^ | |
|  | **Activation of immune response** | All | -0.090 | 0.381 | 0.279 | 0.008 | | 0.011 | |
|  |  | < 80 | 0.052 | 0.708 | 0.105 | 0.458 | | 0.426 | |
|  |  | ≥ 80 | -0.284 | 0.071 | 0.519 | 0.001 | | 0.003 | |
| Key: *APOE*, Apolipoprotein E; PRS, Polygenic Risk Score; p_T,_ p-value with threshold T≤0.5; excl., excluding; incl., including; β, beta coefficient (effect size);  P, P-value; AD, Alzheimer’s Disease; APP, Amyloid Precursor Protein ^¥^ *APOE* region: chromosome 19:44.4Mb 19:46.5Mb  Logistic regression was performed. The models were adjusted for PCs, participants’ age at last assessment and sex. | | | | | | | | | |

**Supplementary Table 3**

Association results between disease status and Alzheimer’s disease pathway PRS in ADNI data – standard errors (SE) and areas under the curve (AUC) results

| **Phenotype** | **Pathway** | **Age** | ***APOE* region^¥^** | | **PRS (excl. *APOE* region)**  **(p_T_**≤**0.5)** | | ***APOE* + PRS**  **(p_T_**≤**0.5)** |
| --- | --- | --- | --- | --- | --- | --- | --- |
|  |  |  | **SE** | **AUC** | **SE** | **AUC** | **AUC** |
| **AD**  **(latest assessment**) | **Protein-lipid complex assembly** | All | 0.121 | 0.711 | 0.101 | 0.512 | 0.704 |
|  |  | < 80 | 0.183 | 0.760 | 0.140 | 0.509 | 0.735 |
|  |  | ≥ 80 | 0.163 | 0.637 | 0.148 | 0.541 | 0.658 |
|  | **Regulation of beta-amyloid formation** | All | 0.121 | 0.687 | 0.102 | 0.552 | 0.697 |
|  |  | < 80 | 0.185 | 0.749 | 0.142 | 0.544 | 0.755 |
|  |  | ≥ 80 | 0.158 | 0.579 | 0.148 | 0.547 | 0.618 |
|  | **Protein-lipid complex** | All | 0.120 | 0.705 | 0.103 | 0.577 | 0.710 |
|  |  | < 80 | 0.184 | 0.760 | 0.143 | 0.569 | 0.756 |
|  |  | ≥ 80 | 0.161 | 0.623 | 0.153 | 0.608 | 0.653 |
|  | **Regulation of APP catabolic process** | All | 0.121 | 0.687 | 0.102 | 0.547 | 0.696 |
|  |  | < 80 | 0.185 | 0.749 | 0.142 | 0.540 | 0.755 |
|  |  | ≥ 80 | 0.158 | 0.579 | 0.148 | 0.543 | 0.623 |
|  | **Tau protein binding** | All | 0.121 | 0.692 | 0.102 | 0.546 | 0.695 |
|  |  | < 80 | 0.186 | 0.760 | 0.140 | 0.547 | 0.768 |
|  |  | ≥ 80 | 0.157 | 0.581 | 0.149 | 0.544 | 0.598 |
|  | **Reverse cholesterol transport** | All | 0.121 | 0.708 | 0.101 | 0.523 | 0.698 |
|  |  | < 80 | 0.183 | 0.761 | 0.140 | 0.510 | 0.724 |
|  |  | ≥ 80 | 0.163 | 0.631 | 0.148 | 0.528 | 0.655 |
|  | **Protein-lipid complex subunit organization** | All | 0.121 | 0.711 | 0.101 | 0.510 | 0.711 |
|  |  | < 80 | 0.183 | 0.760 | 0.140 | 0.515 | 0.760 |
|  |  | ≥ 80 | 0.163 | 0.637 | 0.147 | 0.504 | 0.647 |
|  | **Plasma lipoprotein particle assembly** | All | 0.121 | 0.687 | 0.102 | 0.540 | 0.688 |
|  |  | < 80 | 0.185 | 0.749 | 0.141 | 0.537 | 0.753 |
|  |  | ≥ 80 | 0.158 | 0.579 | 0.149 | 0.551 | 0.602 |
|  | **Activation of immune response** | All | 0.103 | 0.530 | 0.104 | 0.575 | 0.572 |
|  |  | < 80 | 0.139 | 0.511 | 0.141 | 0.527 | 0.532 |
|  |  | ≥ 80 | 0.157 | 0.575 | 0.160 | 0.635 | 0.622 |
| Key: *APOE*, Apolipoprotein E; PRS, Polygenic Risk Score; p_T,_ p-value with threshold T≤0.5; excl., excluding; incl., including; se, standard error; AUC, Area Under the Curve; P, P-value; AD, Alzheimer’s Disease; APP, Amyloid Precursor Protein. ^¥^ *APOE* region: chromosome 19:44.4Mb 19:46.5Mb  Logistic regression was performed. The models were adjusted for PCs, participants’ age at last assessment and sex. | | | | | | | |

**Supplementary Table 4**

Association results between disease status and Alzheimer’s disease pathway PRS in the UK Biobank data – effect sizes (β) and p-values (P)

| **Phenotype** | **Pathway** | **Age** | ***APOE* region^¥^** | | **PRS (excl. *APOE* region)**  **(p_T_**≤**0.5)** | | ***APOE* + PRS**  **(p_T_**≤**0.5)** | |  |
| --- | --- | --- | --- | --- | --- | --- | --- | --- | --- |
|  |  |  | **β** | **P** | **β** | **P** | | **P** | |
| **Parental**  **AD** | **Protein-lipid complex assembly** | All | 0.230 | <1x10^-350^ | 0.026 | 1.3x10^-8^ | | <1x10^-350^ | |
|  |  | < 80 | 0.320 | <1x10^-350^ | 0.019 | 0.029 | | <1x10^-350^ | |
|  |  | ≥ 80 | 0.197 | <1x10^-350^ | 0.029 | 1.1x10^-7^ | | <1x10^-350^ | |
|  | **Regulation of beta-amyloid formation** | All | 0.219 | <1x10^-350^ | 0.033 | 1.2x10^-12^ | | <1x10^-350^ | |
|  |  | < 80 | 0.302 | <1x10^-350^ | 0.026 | 0.0038 | | 4.3x10^-269^ | |
|  |  | ≥ 80 | 0.188 | <1x10^-350^ | 0.036 | 4.8x10^-11^ | | 1.6x10^-319^ | |
|  | **Protein-lipid complex** | All | 0.228 | <1x10^-350^ | 0.014 | 0.0021 | | <1x10^-350^ | |
|  |  | < 80 | 0.318 | <1x10^-350^ | 0.018 | 0.038 | | <1x10^-350^ | |
|  |  | ≥ 80 | 0.194 | <1x10^-350^ | 0.013 | 0.014 | | 3.5x10^-317^ | |
|  | **Regulation of APP catabolic process** | All | 0.219 | <1x10^-350^ | 0.025 | 7.5x10^-8^ | | <1x10^-350^ | |
|  |  | < 80 | 0.302 | <1x10^-350^ | 0.027 | 0.0023 | | <1x10^-350^ | |
|  |  | ≥ 80 | 0.188 | <1x10^-350^ | 0.025 | 3.8x10^-6^ | | 1.3x10^-314^ | |
|  | **Tau protein binding** | All | 0.219 | <1x10^-350^ | 0.020 | 2.0x10^-5^ | | <1x10^-350^ | |
|  |  | < 80 | 0.303 | <1x10^-350^ | 0.031 | 0.0005 | | <1x10^-350^ | |
|  |  | ≥ 80 | 0.188 | <1x10^-350^ | 0.016 | 0.0028 | | 3.3x10^-310^ | |
|  | **Reverse cholesterol transport** | All | 0.229 | <1x10^-350^ | 0.033 | 1.9x10^-12^ | | <1x10^-350^ | |
|  |  | < 80 | 0.320 | <1x10^-350^ | 0.035 | 9.6x10^-5^ | | <1x10^-350^ | |
|  |  | ≥ 80 | 0.195 | <1x10^-350^ | 0.030 | 1.9x10^-8^ | | 4.0x10^-323^ | |
|  | **Protein-lipid complex subunit organization** | All | 0.230 | <1x10^-350^ | 0.021 | 4.6x10^-6^ | | <1x10^-350^ | |
|  |  | < 80 | 0.321 | <1x10^-350^ | 0.015 | 0.092 | | <1x10^-350^ | |
|  |  | ≥ 80 | 0.196 | <1x10^-350^ | 0.023 | 1.4x10^-5^ | | <1x10^-350^ | |
|  | **Plasma lipoprotein particle assembly** | All | 0.219 | <1x10^-350^ | 0.025 | 4.9x10^-8^ | | <1x10^-350^ | |
|  |  | < 80 | 0.302 | <1x10^-350^ | 0.022 | 0.0142 | | <1x10^-350^ | |
|  |  | ≥ 80 | 0.188 | <1x10^-350^ | 0.027 | 6.6x10^-7^ | | 2.7x10^-315^ | |
|  | **Activation of immune response** | All | 0.058 | 2.2x10^-38^ | 0.013 | 0.0068 | | 6.4x10^-38^ | |
|  |  | < 80 | 0.084 | 1.4x10^-23^ | 0.017 | 0.060 | | 1.7x10^-22^ | |
|  |  | ≥ 80 | 0.050 | 2.2x10^-21^ | 0.010 | 0.073 | | 1.3x10^-20^ | |
| Key: APOE, Apolipoprotein E; PRS, Polygenic Risk Score; p_T,_ p-value with threshold T≤0.5; excl., excluding; incl., including; β, beta coefficient (effect size);  P, P-value; AD, Alzheimer’s Disease; APP, Amyloid Precursor Protein ^¥^ *APOE* region: chromosome 19:44.4Mb 19:46.5Mb  Poisson regression was performed. The models were adjusted for PCs, parental age and participants’ sex. | | | | | | | | | |

**Supplementary Table 5**

Association results between disease status and Alzheimer’s disease pathway PRS in the UK Biobank data – standard errors (SE) and areas under the curve (AUC) results

| **Phenotype** | **Pathway** | **Age** | ***APOE* region^¥^** | | **PRS (excl. *APOE* region)**  **(p_T_**≤**0.5)** | | ***APOE* + PRS**  **(p_T_**≤**0.5)** |
| --- | --- | --- | --- | --- | --- | --- | --- |
|  |  |  | **SE** | **AUC** | **SE** | **AUC** | **AUC** |
| **AD**  **(latest assessment**) | **Protein-lipid complex assembly** | All | 0.004 | 0.573 | 0.005 | 0.509 | 0.572 |
|  |  | < 80 | 0.008 | 0.593 | 0.009 | 0.508 | 0.593 |
|  |  | ≥ 80 | 0.005 | 0.572 | 0.005 | 0.512 | 0.573 |
|  | **Regulation of beta-amyloid formation** | All | 0.004 | 0.592 | 0.005 | 0.511 | 0.579 |
|  |  | < 80 | 0.007 | 0.601 | 0.009 | 0.509 | 0.600 |
|  |  | ≥ 80 | 0.005 | 0.577 | 0.005 | 0.514 | 0.578 |
|  | **Protein-lipid complex** | All | 0.004 | 0.570 | 0.005 | 0.506 | 0.569 |
|  |  | < 80 | 0.008 | 0.591 | 0.009 | 0.508 | 0.592 |
|  |  | ≥ 80 | 0.005 | 0.568 | 0.005 | 0.507 | 0.568 |
|  | **Regulation of APP catabolic process** | All | 0.004 | 0.592 | 0.005 | 0.509 | 0.580 |
|  |  | < 80 | 0.007 | 0.601 | 0.009 | 0.510 | 0.600 |
|  |  | ≥ 80 | 0.005 | 0.577 | 0.005 | 0.511 | 0.576 |
|  | **Tau protein binding** | All | 0.004 | 0.577 | 0.005 | 0.507 | 0.574 |
|  |  | < 80 | 0.007 | 0.595 | 0.009 | 0.510 | 0.597 |
|  |  | ≥ 80 | 0.005 | 0.572 | 0.005 | 0.506 | 0.572 |
|  | **Reverse cholesterol transport** | All | 0.004 | 0.569 | 0.005 | 0.511 | 0.570 |
|  |  | < 80 | 0.008 | 0.591 | 0.009 | 0.512 | 0.592 |
|  |  | ≥ 80 | 0.005 | 0.568 | 0.005 | 0.512 | 0.570 |
|  | **Protein-lipid complex subunit organization** | All | 0.004 | 0.573 | 0.005 | 0.507 | 0.570 |
|  |  | < 80 | 0.008 | 0.592 | 0.009 | 0.506 | 0.592 |
|  |  | ≥ 80 | 0.005 | 0.569 | 0.005 | 0.510 | 0.570 |
|  | **Plasma lipoprotein particle assembly** | All | 0.004 | 0.592 | 0.005 | 0.509 | 0.579 |
|  |  | < 80 | 0.007 | 0.601 | 0.009 | 0.508 | 0.600 |
|  |  | ≥ 80 | 0.005 | 0.577 | 0.005 | 0.511 | 0.577 |
|  | **Activation of immune response** | All | 0.004 | 0.516 | 0.005 | 0.504 | 0.517 |
|  |  | < 80 | 0.008 | 0.520 | 0.009 | 0.504 | 0.522 |
|  |  | ≥ 80 | 0.005 | 0.517 | 0.005 | 0.504 | 0.518 |
| Key: APOE, Apolipoprotein E; PRS, Polygenic Risk Score; p_T,_ p-value with threshold T≤0.5; excl., excluding; incl., including; SE, standard error; AUC, Area Under the Curve; P, P-value; AD, Alzheimer’s Disease; APP, Amyloid Precursor Protein  ^¥^ *APOE* region: chromosome 19:44.4Mb 19:46.5Mb  Poisson regression was performed. The models were adjusted for PCs, parental age and participants’ sex. | | | | | | | |

**References**

Kunkle, B.W., Grenier-Boley, B., Sims, R., Bis, J.C., Damotte, V., Naj, A.C., Boland, A., Vronskaya, M., van der Lee, S.J., Amlie-Wolf, A., Bellenguez, C., Frizatti, A., Chouraki, V., Martin, E.R., Sleegers, K., Badarinarayan, N., Jakobsdottir, J., Hamilton-Nelson, K.L., Moreno-Grau, S., Olaso, R., Raybould, R., Chen, Y., Kuzma, A.B., Hiltunen, M., Morgan, T., Ahmad, S., Vardarajan, B.N., Epelbaum, J., Hoffmann, P., Boada, M., Beecham, G.W., Garnier, J.G., Harold, D., Fitzpatrick, A.L., Valladares, O., Moutet, M.L., Gerrish, A., Smith, A.V., Qu, L., Bacq, D., Denning, N., Jian, X., Zhao, Y., Del Zompo, M., Fox, N.C., Choi, S.H., Mateo, I., Hughes, J.T., Adams, H.H., Malamon, J., Sanchez-Garcia, F., Patel, Y., Brody, J.A., Dombroski, B.A., Naranjo, M.C.D., Daniilidou, M., Eiriksdottir, G., Mukherjee, S., Wallon, D., Uphill, J., Aspelund, T., Cantwell, L.B., Garzia, F., Galimberti, D., Hofer, E., Butkiewicz, M., Fin, B., Scarpini, E., Sarnowski, C., Bush, W.S., Meslage, S., Kornhuber, J., White, C.C., Song, Y., Barber, R.C., Engelborghs, S., Sordon, S., Voijnovic, D., Adams, P.M., Vandenberghe, R., Mayhaus, M., Cupples, L.A., Albert, M.S., De Deyn, P.P., Gu, W., Himali, J.J., Beekly, D., Squassina, A., Hartmann, A.M., Orellana, A., Blacker, D., Rodriguez-Rodriguez, E., Lovestone, S., Garcia, M.E., Doody, R.S., Munoz-Fernadez, C., Sussams, R., Lin, H., Fairchild, T.J., Benito, Y.A., Holmes, C., Karamujić-Čomić, H., Frosch, M.P., Thonberg, H., Maier, W., Roschupkin, G., Ghetti, B., Giedraitis, V., Kawalia, A., Li, S., Huebinger, R.M., Kilander, L., Moebus, S., Hernández, I., Kamboh, M.I., Brundin, R., Turton, J., Yang, Q., Katz, M.J., Concari, L., Lord, J., Beiser, A.S., Keene, C.D., Helisalmi, S., Kloszewska, I., Kukull, W.A., Koivisto, A.M., Lynch, A., Tarraga, L., Larson, E.B., Haapasalo, A., Lawlor, B., Mosley, T.H., Lipton, R.B., Solfrizzi, V., Gill, M., Longstreth, W.T., Montine, T.J., Frisardi, V., Diez-Fairen, M., Rivadeneira, F., Petersen, R.C., Deramecourt, V., Alvarez, I., Salani, F., Ciaramella, A., Boerwinkle, E., Reiman, E.M., Fievet, N., Rotter, J.I., Reisch, J.S., Hanon, O., Cupidi, C., Andre Uitterlinden, A.G., Royall, D.R., Dufouil, C., Maletta, R.G., de Rojas, I., Sano, M., Brice, A., Cecchetti, R., George-Hyslop, P.S., Ritchie, K., Tsolaki, M., Tsuang, D.W., Dubois, B., Craig, D., Wu, C.K., Soininen, H., Avramidou, D., Albin, R.L., Fratiglioni, L., Germanou, A., Apostolova, L.G., Keller, L., Koutroumani, M., Arnold, S.E., Panza, F., Gkatzima, O., Asthana, S., Hannequin, D., Whitehead, P., Atwood, C.S., Caffarra, P., Hampel, H., Quintela, I., Carracedo, Á., Lannfelt, L., Rubinsztein, D.C., Barnes, L.L., Pasquier, F., Frölich, L., Barral, S., McGuinness, B., Beach, T.G., Johnston, J.A., Becker, J.T., Passmore, P., Bigio, E.H., Schott, J.M., Bird, T.D., Warren, J.D., Boeve, B.F., Lupton, M.K., Bowen, J.D., Proitsi, P., Boxer, A., Powell, J.F., Burke, J.R., Kauwe, J.S.K., Burns, J.M., Mancuso, M., Buxbaum, J.D., Bonuccelli, U., Cairns, N.J., McQuillin, A., Cao, C., Livingston, G., Carlson, C.S., Bass, N.J., Carlsson, C.M., Hardy, J., Carney, R.M., Bras, J., Carrasquillo, M.M., Guerreiro, R., Allen, M., Chui, H.C., Fisher, E., Masullo, C., Crocco, E.A., DeCarli, C., Bisceglio, G., Dick, M., Ma, L., Duara, R., Graff-Radford, N.R., Evans, D.A., Hodges, A., Faber, K.M., Scherer, M., Fallon, K.B., Riemenschneider, M., Fardo, D.W., Heun, R., Farlow, M.R., Kölsch, H., Ferris, S., Leber, M., Foroud, T.M., Heuser, I., Galasko, D.R., Giegling, I., Gearing, M., Hüll, M., Geschwind, D.H., Gilbert, J.R., Morris, J., Green, R.C., Mayo, K., Growdon, J.H., Feulner, T., Hamilton, R.L., Harrell, L.E., Drichel, D., Honig, L.S., Cushion, T.D., Huentelman, M.J., Hollingworth, P., Hulette, C.M., Hyman, B.T., Marshall, R., Jarvik, G.P., Meggy, A., Abner, E., Menzies, G.E., Jin, L.W., Leonenko, G., Real, L.M., Jun, G.R., Baldwin, C.T., Grozeva, D., Karydas, A., Russo, G., Kaye, J.A., Kim, R., Jessen, F., Kowall, N.W., Vellas, B., Kramer, J.H., Vardy, E., LaFerla, F.M., Jöckel, K.H., Lah, J.J., Dichgans, M., Leverenz, J.B., Mann, D., Levey, A.I., Pickering-Brown, S., Lieberman, A.P., Klopp, N., Lunetta, K.L., Wichmann, H.E., Lyketsos, C.G., Morgan, K., Marson, D.C., Brown, K., Martiniuk, F., Medway, C., Mash, D.C., Nöthen, M.M., Masliah, E., Hooper, N.M., McCormick, W.C., Daniele, A., McCurry, S.M., Bayer, A., McDavid, A.N., Gallacher, J., McKee, A.C., van den Bussche, H., Mesulam, M., Brayne, C., Miller, B.L., Riedel-Heller, S., Miller, C.A., Miller, J.W., Al-Chalabi, A., Morris, J.C., Shaw, C.E., Myers, A.J., Wiltfang, J., O'Bryant, S., Olichney, J.M., Alvarez, V., Parisi, J.E., Singleton, A.B., Paulson, H.L., Collinge, J., Perry, W.R., Mead, S., Peskind, E., Cribbs, D.H., Rossor, M., Pierce, A., Ryan, N.S., Poon, W.W., Nacmias, B., Potter, H., Sorbi, S., Quinn, J.F., Sacchinelli, E., Raj, A., Spalletta, G., Raskind, M., Caltagirone, C., Bossù, P., Orfei, M.D., Reisberg, B., Clarke, R., Reitz, C., Smith, A.D., Ringman, J.M., Warden, D., Roberson, E.D., Wilcock, G., Rogaeva, E., Bruni, A.C., Rosen, H.J., Gallo, M., Rosenberg, R.N., Ben-Shlomo, Y., Sager, M.A., Mecocci, P., Saykin, A.J., Pastor, P., Cuccaro, M.L., Vance, J.M., Schneider, J.A., Schneider, L.S., Slifer, S., Seeley, W.W., Smith, A.G., Sonnen, J.A., Spina, S., Stern, R.A., Swerdlow, R.H., Tang, M., Tanzi, R.E., Trojanowski, J.Q., Troncoso, J.C., Van Deerlin, V.M., Van Eldik, L.J., Vinters, H.V., Vonsattel, J.P., Weintraub, S., Welsh-Bohmer, K.A., Wilhelmsen, K.C., Williamson, J., Wingo, T.S., Woltjer, R.L., Wright, C.B., Yu, C.E., Yu, L., Saba, Y., Pilotto, A., Bullido, M.J., Peters, O., Crane, P.K., Bennett, D., Bosco, P., Coto, E., Boccardi, V., De Jager, P.L., Lleo, A., Warner, N., Lopez, O.L., Ingelsson, M., Deloukas, P., Cruchaga, C., Graff, C., Gwilliam, R., Fornage, M., Goate, A.M., Sanchez-Juan, P., Kehoe, P.G., Amin, N., Ertekin-Taner, N., Berr, C., Debette, S., Love, S., Launer, L.J., Younkin, S.G., Dartigues, J.F., Corcoran, C., Ikram, M.A., Dickson, D.W., Nicolas, G., Campion, D., Tschanz, J., Schmidt, H., Hakonarson, H., Clarimon, J., Munger, R., Schmidt, R., Farrer, L.A., Van Broeckhoven, C., C O'Donovan, M., DeStefano, A.L., Jones, L., Haines, J.L., Deleuze, J.F., Owen, M.J., Gudnason, V., Mayeux, R., Escott-Price, V., Psaty, B.M., Ramirez, A., Wang, L.S., Ruiz, A., van Duijn, C.M., Holmans, P.A., Seshadri, S., Williams, J., Amouyel, P., Schellenberg, G.D., Lambert, J.C., Pericak-Vance, M.A., Alzheimer Disease Genetics Consortium (ADGC), European Alzheimer’s Disease Initiative (EADI), Cohorts for Heart and Aging Research in Genomic Epidemiology Consortium (CHARGE), Genetic and Environmental Risk in AD/Defining Genetic, P.l.a.E.R.f.A.s.D.C.G.P., 2019. Genetic meta-analysis of diagnosed Alzheimer's disease identifies new risk loci and implicates Aβ, tau, immunity and lipid processing. Nat Genet 51(3), 414-430.
